# The Role of Syntrophic Associations in Sustaining Anaerobic Mineralization of Chlorinated Organic Compounds

**DOI:** 10.1289/ehp.6933

**Published:** 2004-12-08

**Authors:** Jennifer G. Becker, Gina Berardesco, Bruce E. Rittmann, David A. Stahl

**Affiliations:** Department of Civil and Environmental Engineering, Northwestern University, Evanston, Illinois, USA

**Keywords:** 2-chlorophenol, 3-chlorobenzoate, benzoate, bioremediation, microbial communities, oligonucleotide probes, reductive dechlorination, ribosomal RNA, syntrophic associations, *Syntrophus*

## Abstract

Stable associations of syntrophic fermentative organisms and populations that consume fermentation products play key roles in the anaerobic biodegradation of chlorinated organic contaminants. The involvement of these syntrophic populations is essential for mineralization of chlorinated aromatic compounds under methanogenic conditions. The fermentative production of low levels of hydrogen (H_2_) can also be used to selectively deliver a limiting electron donor to dehalogenating organisms and achieve complete dehalogenation of chlorinated aliphatic contaminants such as tetrachloroethene. Thus, tracking the abundance of syntrophically coupled populations should aid in the development and monitoring of sustainable bioremediation strategies. In this study, two complementary nucleic acid–based methods were used to identify and assess relative changes or differences in the abundance of potentially important populations in complex anaerobic microbial communities that mineralized chlorinated aromatic compounds. Population dynamics were related to the consumption and production of key metabolic substrates, intermediates, and products. *Syntrophus*-like populations were detected in 3-chlorobenzoate–degrading communities derived from sediment or sludge digesters. In the presence of H_2_-consuming populations, characterized *Syntrophus* species ferment benzoate, a central intermediate in the anaerobic metabolism of 3-chlorobenzoate and 2-chlorophenol. A DNA probe that targeted characterized *Syntrophus* species was developed and used to quantify rRNA extracted from the 3-chlorobenzoate– and 2-chlorophenol–degrading communities. The level of rRNA targeted by the *Syntrophus*-specific probe tracked with the formation of benzoate during metabolism of the parent compounds. Hybridizations with an Archaea-specific probe and/or measurement of methane production demonstrated that methanogens directly benefited from the influx of benzoate-derived electron donors, and the activities of *Syntrophus*-like and methanogenic populations in the contaminant-degrading communities were closely linked.

Microbiologists now recognize that the greater part of microbial diversity remains to be described, primarily because culture-based methods cannot provide an adequate census of community composition (Amann et al. 1995). This has severely restricted studies in both basic and applied microbial ecology. For example, researchers understand little about the complex and dynamic microbial processes associated with transformation of pollutants in the environment. Understanding how populations within microbial communities respond to and metabolize contaminants is key to predicting contaminant fate at sites undergoing intrinsic bioremediation and to optimizing engineered bioremediation approaches. The advent of explicit molecular methods has improved our ability to describe the microbial ecology of contaminated and other environments (Liu and Stahl 2002). However, linking changes in population structure with system-level processes remains challenging when the composition of the community is not known a priori and the function of individual populations cannot be studied in defined cultures.

In particular, little is known about the composition of anaerobic microbial communities that degrade chlorinated organic compounds *in situ*. Culture-based description of these communities is hampered by at least two factors. First, syntrophic population associations are a hallmark of anaerobic microbial communities in general. The interdependence of syntrophic partners severely complicates their cultivation (Schink 1997). Second, the enrichment of bacteria that utilize chlorinated organic compounds such as tetrachloroethene (perchloroethylene, PCE) as the terminal electron acceptor in metabolic processes (i.e., dehalorespiration) can pose numerous challenges. These challenges include providing low-solubility chlorinated substrates (e.g., PCE) at levels that are nontoxic yet adequate to sustain growth and meeting the specific nutritional requirements of the dehalorespiring organisms (Holliger and Schumacher 1994).

This lack of information is unfortunate because the chlorinated ethenes PCE and trichloroethene are among the most commonly detected groundwater contaminants (Council on Environmental Quality 1981). Complete biodegradation of these compounds at contaminated sites is often limited by the availability of a suitable electron donor (He et al. 2002). A number of microbial populations that use PCE as the terminal electron acceptor in dehalorespiration couple the reduction of chlorinated ethenes to the oxidation of hydrogen (H_2_). Thus, although the direct involvement of organic electron donors, especially acetate, in dehalorespiration has been demonstrated at some contaminated sites (He et al. 2002), it is generally believed that H_2_ serves as the ultimate electron donor for reduction of PCE in most cases (Fennell et al. 1997; Yang and McCarty 1998). However, sustaining degradation of chlorinated ethenes through the delivery of H_2_ in engineered *in situ* bioremediation scenarios is complicated by the presence of a variety of hydrogenotrophic populations that use nonchlorinated electron acceptors, such as sulfate and carbon dioxide (CO_2_), in contaminated anaerobic environments and the competition of these populations with PCE-degrading organisms for H_2_.

One strategy for enhancing selective delivery of H_2_ to dehalorespiring populations involves providing H_2_ through the addition of certain fermentable substrates such as butyrate or propionate (Fennell et al. 1997). Fermentation of butyrate under typical culture conditions occurs slowly and is thermo-dynamically feasible only at H_2_ partial pressures of 10–3.5 atm or less. Even lower H_2_ levels are required to sustain fermentation of propionate. Dehalorespiring and other hydrogenotrophic populations in anaerobic environments maintain H_2_ levels low enough to allow fermentation of a wide range of organic substrates to proceed. However, production of H_2_ through the slow syntrophic fermentation of butyrate and similar compounds appears to favor dechlorination over other hydrogenotrophic processes. Thus, being able to track the abundance of syntrophic H_2_-producing and H_2_-consuming populations should be useful when developing and monitoring sustainable engineering strategies for remediation of chloroethene-contaminated sites.

The ability to track syntrophic populations that produce and consume H_2_ (and/or acetate) in complex anaerobic microbial communities that degrade chlorinated aromatic compounds is also useful because of the key roles that these populations play in sustaining biodegradation of the parent compounds (Becker et al. 2001). Therefore, we used two model microbial communities to study the degradation of 3-chlorobenzoate (3-CB) and 2-chlorophenol (2-CP). The communities were maintained in batch laboratory systems derived from two different anaerobic habitats: lake sediment and municipal wastewater sludge. Two complementary nucleic acid–based methods were used to evaluate population changes relative to substrate production and use in these systems. The complementary analyses clearly resolved the role of syntrophic associations in the degradation of both chlorinated compounds.

## Materials and Methods

### Source of inocula.

The 3-CB– and 2-CP–degrading communities were derived from environmental inocula obtained from anaerobic lake sediment or municipal wastewater sludge digesters. Sediment was obtained from the previously described (MacGregor et al. 1997) Fox Point sampling site in Lake Michigan during two sampling events. Anaerobic sediment was collected using a box corer and transferred to canning jars, which were filled to capacity and stored at 4°C until used (within ~ 3 months). Sediment collected during the first event provided the inoculum for a preliminary microcosm experiment conducted with 3-CB, whereas the inoculum used in mesocosm-scale experiments involving 2-CP or 3-CB was derived from sediment collected nearly 2 years later. A mixture of primary and secondary digester sludge was obtained from the North Shore Sanitary District’s Clavey Road Wastewater Treatment Plant in Highland Park, Illinois. After some material was flushed from a sampling line, canning jars were filled until they overflowed and stored at 4°C until used (within 24 hr). The digester contents were used as the inoculum in a second mesocosm-scale experiment involving 3-CB.

### Establishment of cultures.

The sediment and digester inocula were diluted in anaerobic mineral medium (1:9, vol/vol), and the cultures were maintained in microcosms and mesocosms comprising 160-mL and 2-L glass vessels, respectively, with serum bottle closures, using the methods described by Becker et al. (1999). Culture preparation, sampling, and amendments were performed using strict anaerobic techniques based on the methods described by Miller and Wolin (1974). The anaerobic mineral medium used in the sediment experiments has been described previously (Freedman and Gossett 1989). The medium used to dilute the digester inoculum had a slightly different formulation and contained (per liter): 0.5 g NH_4_Cl, 0.4 g K_2_HPO_4_, 0.1 g MgCl_2_ × 6H_2_O, 0.001 g resazurin, 0.5 g Na_2_S × 9H_2_O, 0.1 g CaCl_2_ × 2H_2_O, 4 g NaHCO_3_, 0.05 g yeast extract, and 10 mL trace metal solution. The trace metal solution was modified from Tanner (1997) and contained (per liter): 1 g MnCl_2_ × 4H_2_O, 0.2 g CoCl_2_ × 6H_2_O, 0.2 g ZnSO_4_ × 7H_2_O, 0.019 g H_3_BO_4_, 0.02 g NiCl_2_ × 6H_2_O, 0.02 g Na_2_MoO_4_ × 2H_2_O, 0.8 g Fe(NH_4_)_2_(SO_4_)_2_, 0.02 g CuCl_2_ × 2H_2_O, 0.02 g Na_2_SeO_3_, 0.02 g Na_2_WO_4_, and 2 g nitriloacetic acid. Cultures were maintained under a headspace of 30% CO_2_:70% N_2_ (vol/vol). The initial ratio of headspace volume to slurry-phase volume in all culture vessels was 3:5 (vol/vol). All cultures and controls were incubated statically at 30°C.

### Microcosm-scale biodegradation experiment.

A preliminary experiment was conducted using 160-mL serum bottle microcosms inoculated with sediment and amended with 3-CB. Sediment microcosms were prepared in triplicate and amended with 3-CB (99+%; Aldrich, Milwaukee, WI) at an initial concentration of 200 μM. Duplicate sterile controls were prepared in the same way except that the sediment slurries were autoclaved for 1 hr on each of 2 consecutive days before being amended with 3-CB. The microcosms and sterile controls were sampled regularly for analysis of 3-CB, which was resupplied to the microcosms whenever it was depleted. Slurry samples were periodically taken from the microcosms for analysis of community rDNA using denaturing gradient gel electrophoresis (DGGE).

### Mesocosm-scale biodegradation experiments.

Mesocosm-scale biodegradation experiments were conducted using 2-L culture vessels inoculated with sediment or digester sludge, amended with either 3-CB (sediment and digester sludge) or 2-CP (sediment), and established and maintained using aseptic techniques. Viable 2-L mesocosms were amended with 3-CB or 2-CP (99+%; Aldrich) at an initial concentration of 200 μM. No-substrate controls (2 L) were maintained for each experiment and did not receive any chlorinated substrate additions. Sterile controls (2 L) were prepared by autoclaving the inocula for 1 hr on 3 consecutive days before combining them with sterile medium and 3-CB (digester inoculum) or 3-CB plus 2-CP (sediment inoculum). The mesocosms and control reactors were sampled at approximately 1-week intervals. For each experiment, duplicate or triplicate microcosms (160-mL) were prepared in the same way as the viable 2-CP– and 3-CB–amended mesocosms and periodically sampled to evaluate the reproducibility of the nucleic acid and chemical data obtained with the mesocosms (Becker 1998). Aromatic substrates and metabolites were monitored in all reactors amended with 3-CB and/or 2-CP. rRNA was extracted from all viable reactors. All other analyses were performed only on the viable 2-L reactors. In the interest of brevity, only the results obtained with the mesocosms are reported here.

### Benzoate perturbation experiment.

At the conclusion of the mesocosm-scale biodegradation experiment involving the digester sludge inoculum, duplicate 50-mL aliquots of slurry were aseptically removed from the 3-CB–amended mesocosm and transferred to 160-mL serum bottles. Except for the difference in the ratio of headspace volume to slurry volume, the transferred 3-CB–degrading digester cultures were maintained as described above. The cultures were amended with a sterile, deoxygenated stock solution of benzoate, resulting in an initial concentration of 13.8 mM, and regularly sampled for analysis of benzoate.

### Analytical methods.

Reverse-phase high-performance liquid chromatography with diode array detection was used to quantify chlorinated aromatic substrates and metabolites, as previously described (Becker et al. 1999). Headspace H_2_ and methane (CH_4_) concentrations were determined using gas chromatography and reduction gas and flame ionization detectors, respectively, according to the methods described by Becker et al. (2001).

### DNA extraction, amplification, separation, and sequencing.

DNA was isolated from sediment and digester slurry samples using a modification of the freeze-thaw and lysozyme treatments, phenol–chloroform extraction, and ethanol precipitation described by Tsai and Olson (1991). Each slurry sample (1 mL) was transferred to a plastic centrifuge tube (14 mL; Sarstedt, Inc., Nümbrecht, Germany) and frozen (−20°C) until cell lysis and extraction and precipitation of DNA with ethanol was implemented. Extracted DNA was diluted (1:10) and amplified using the bacteria-specific primers GM5F-GC and S-*-Univ-0907-b-A-20 and the polymerase chain reaction (PCR) procedure described by Muyzer et al. (1993) with slight modification. The GM5F-GC primer contains a 40-bp GC clamp to introduce a high-melting-point domain into the amplified fragment. Together, primers GM5F-GC and S-*-Univ-0907-b-A-20 amplify bacterial small-subunit (SSU) rDNA in the region corresponding to positions 357–907 in *Escherichia coli* to produce a 550-bp fragment (not including the GC-clamp). The 50-μL reaction mixture consisted of 100–300 ng (2–5 μL) template DNA;10 × Taq polymerase buffer [500 mM KCl, 15 mM MgCl_2_, 100 mM Tris-HCl (Pharmacia, Piscataway, NJ); 5 μL], 20 pmol each of forward and reverse primers, 0.2 mM solution of deoxyribonucleoside triphosphates, 1.5 U Taq (Pharmacia), and 500 ng/μL bovine serum albumin (Idaho Technologies, Idaho Falls, ID). Touchdown PCR amplifications were typically performed in a thermocycler (model 200; MJ Research, Inc., Watertown, MA), as described by Becker et al. (2001). Separation of the amplified bacterial community DNA via DGGE was performed using the Bio-Rad D GENE system (Bio-Rad, Hercules, CA) with slight modifications to the manufacturer’s instructions. The PCR products were loaded onto a 6% (wt/vol) polyacrylamide gel containing a denaturing gradient of urea and formamide. The gel was run for 4 hr at 200 V at 60°C. After electrophoresis the gel was stained with ethidium bromide and the image captured with a digital camera (Kaiser Fototechnik GmbH & Co., Buchen, Germany). Bands of interest were excised from a DGGE gel with a razor blade, placed in a micro-centrifuge tube, and incubated at room temperature for 10 min in 10–15 μL of sterile distilled water. This suspension (1–2 μL) was used as template for reamplification, as described above, using primers S-D-Bact-0341-a-S-17 and S-*-Univ-0907-b-A-20. Primer S-D-Bact-0341-a-S-17 is GM4F-GC without the GC-clamp (5′-CCT ACG GGA GGC AGC AG-3′). The resulting PCR product was directly sequenced using a SequiTherm Long-Read kit (Epicenter Technologies, Madison, WI), infrared-labeled primers S-D-Bact-0341-a-S-17 and S-*-Univ-0907-b-A-20 (LI-COR Corp., Lincoln, NE), and an automated DNA sequencer (model 400L; LI-COR Corp.). The resulting sequences were locally aligned using the FASTA3 algorithm (Pearson and Lipman 1988) to identify the best matches. Multiple sequence alignment was needed for development of a group-specific phylogenetic probe and was performed using software available through the Ribosomal Database Project II (Cole et al. 2003). Additional phylogenetic analyses were performed using the PHYLIP software package (version 3.5c; Felsenstein 1993).

### RNA extraction, hybridization, and quantification of extracted RNA.

The mechanical-disruption/phenol–chloroform extraction process of Stahl et al. (1988) was used with previously described modifications (Becker et al. 2001; MacGregor et al. 1997) to extract RNA from sediment and digester slurry samples. RNA slot blotting, probe labeling, prehybridization, hybridization, and washing were performed as previously described (Lin and Stahl 1995; Raskin et al. 1994; Stahl et al. 1988). Samples were transferred to nylon membranes in triplicate, pre-hybridized at 40°C, and washed at 56°C [S-D-Arch-0915-a-A-20; Amann et al. (1990)] or 51°C [S-G-Syn-0424-a-A-18; Becker et al. (2001)]. It was assumed that archaeal rRNA, which was targeted by probe S-D-Arch-0915-a-A-20, was primarily derived from methanogenic species. This is reasonable because previous studies have shown that crenarchaeotal rRNA is a minor component of the Lake Michigan sediment (MacGregor et al. 1997). Probe S-G-Syn-0424-a-A-18 targets members of the genus *Syntrophus* and has been described previously (Becker et al. 2001) but was developed as part of this study. The relative abundance of rRNA that hybridized with the Archaea- and *Syntrophus*-specific probes in a given mesocosm is reported relative to the amount of archaeal and *Syntrophus*-like rRNA, respectively, in the reactor at time zero.

## Results and Discussion

**Figure 1:**
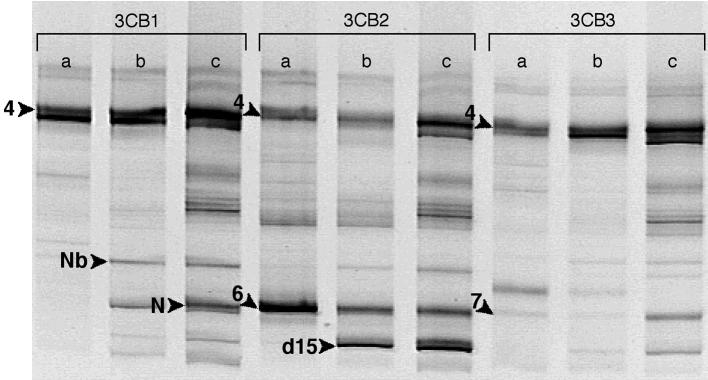
Ethidium bromide–stained DGGE profiles of rDNA PCR fragments derived from DNA extracted from triplicate 3-CB–degrading microcosms inoculated with Lake Michigan sediment (3CB1, 3CB2, and 3CB3) using a 20–70% denaturant gradient gel. The DNA extracts were obtained from the microcosms on the following days: lane a, day 330; lane b, day 500; lane c, day 600. Gel portions that were excised for additional analyses are indicated with arrows plus a letter and/or number. Throughout the text and in Figure 2, these bands are referred to using the microcosm name plus the label shown in Figure 1 (e.g., 3CB1-4). Sequences derived from reamplified DGGE bands were submitted to GenBank (http://www.ncbi.nlm.nih.gov/entrez/query.fcgi?db_nucleotide) with the following accession numbers: 3CB1-4, AY646230; 3CB1-Nb, AY646231; 3CB1-N, AY646232; 3CB2-4, AY646233; 3CB2-6, AY646234; 3CB2-d15, AY646235; 3CB3-4, AY646236; and 3CB3-7, AY646237.

In the preliminary studies of biodegradation of 3-CB in sediment, biodegradation was not observed for approximately 78 days. Thereafter, 3-CB was rapidly degraded in the triplicate microcosms but not in the sterile controls (data not shown). 3-CB was repeatedly degraded after 29 replenishments (~ 200-μM amendments) over an approximately 2-year period. DNA samples were obtained from the microcosms on days 330, 500, and 600 and analyzed using DGGE (Figure 1). In each of the microcosms, the DGGE separation patterns varied over time, which suggests that the structures of the 3-CB–degrading communities were not static, even after degrading repetitive additions of 3-CB. Patterns also differed among microcosms, despite their similar treatment and behavior. However, several DNA fragments migrated to similar positions in the DGGE gel in all three microcosms, even after 500 or 600 days. A number of these bands were unique to the 3-CB–degrading microcosms and did not appear in the DGGE patterns obtained from microcosms that did not adapt to 3-CB (Becker 1998).

Selected bands were excised from the DGGE gel for reamplification and comparative sequencing (Figure 1). One band (position 4) was common to all lanes. The bands in position 4 actually consisted of two DNA fragments that comigrated in the 20–70% denaturant gradient gel. The comigrating fragments could be resolved using a 30–50% denaturant gradient gel (not shown). Each of the other amplified bands corresponded to a single sequence. Sequence lengths ranged from 338 to 467 nucleotides. The sequences contained a maximum of five ambiguities, and most of the sequences contained zero to two ambiguities. Ambiguous sequence regions were not included in similarity determinations.

Sequences 3CB2-4 and 3CB3-4 are closely related to uncultured members of anaerobic communities that dechlorinate 1,2-dichloropropane (SHA-207; Schlotelburg et al. 2000) and trichlorobenzene (SJA-162; von Wintzingerode et al. 1999). 3CB2-4 shares a sequence similarity of 99 and 98% with SHA-207 and SJA-16, respectively. 3CB3-4 shares 97% sequence similarity with SHA-207 and SJA-162. Another fragment from the 3-CB-degrading sediment microcosms (3CB1-Nb) originated from a close relative of *Desulfomonile tiedjei*, which is able to conserve energy via the reductive dehalogenation of 3-CB (DeWeerd et al. 1990). 3CB1-Nb and *D. tiedjei* share > 99% sequence similarity over 429 bp.

The closest cultured relative of both 3CB1-N and 3CB2-d15 is *Moorella* sp. F21 (GenBank accession no. AB086398; http://www.ncbi.nlm.nih.gov/entrez/query.fcgi?db_nucleotide). Strain F21 shares 87 and 91% sequence similarity with 3CB1-N and 3CB2-d15, respectively. 3CB3-7 shares 97% sequence similarity with its closest relative, a clone of an uncultured bacterium from a thermophilic terephthalate-degrading anaerobic sludge (GenBank accession no. AY297966; http://www.ncbi.nlm.nih.gov/entrez/query.fcgi?db_nucleotide).

**Figure 2:**
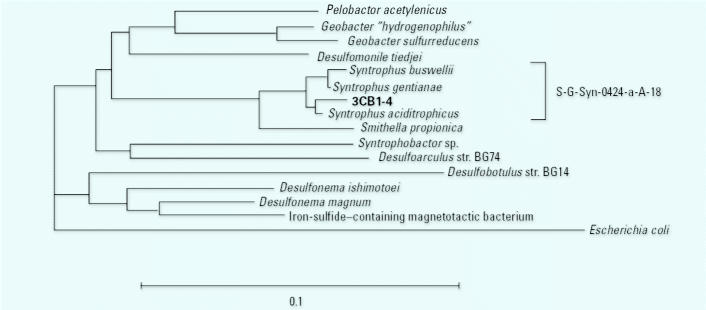
Phylogenetic placement of sequence 3CB1-4, which was derived from a 3-CB–degrading sediment microcosm. The neighbor joining tree is based on 16S rRNA sequence positions 357–907 (*E. coli* numbering). The solid bracket shows the sequences targeted by S-G-Syn-0424-a-A-18, and the scale bar represents the estimated number of base changes per nucleotide. Bold type indicates the sequence was obtained in this study.

Two sequences are closely related to benzoate-degrading syntrophs. Conversion of benzoate to H_2_, acetate, and CO_2_ is thermo-dynamically unfavorable at standard conditions. However, in the presence of hydrogen-consuming populations that maintain low H_2_ partial pressures in anaerobic environments, the fermentation of benzoate to H_2_, acetate, and CO_2_ by syntrophic bacteria is thermodynamically feasible. The closest cultured relatives of 3CB1-4 are the characterized *Syntrophus* species *S. buswellii* (Mountfort et al. 1984), *S. gentianae* (Wallrabenstein et al. 1995), and *S. aciditrophicus* (Jackson et al. 1999) (Figure 2). For example, 3CB1-4 shares 99% sequence similarity with *S. aciditrophicus*. Although fermentation of benzoate to cyclohexane carboxylate and acetate by a pure culture of *S. aciditrophicus* has been observed (Elshahed and McInerney 2001), the characterized *Syntrophus* species appear to grow primarily via syntrophic fermentation of benzoate (Jackson et al. 1999; Mountfort et al. 1984; Wallrabenstein et al. 1995). 3CB2-6 shares 95% sequence similarity with the gram-positive benzoate-degrading syntroph *Sporotomaculum syntrophicum* (Qiu et al. 2003).

Benzoate is a central intermediate in the anaerobic biodegradation of a variety of aromatic compounds, including 3-CB and *ortho*-chlorinated phenols (Heider and Fuchs 1997) and is produced from the reductive dehalogenation of 3-CB. Consumption of H_2_ by a dehalogenating organism (e.g., *Desulfomonile tiedjei*) and/or methanogenic populations creates an environment in which benzoate fermentation is feasible and biodegradation of 3-CB can be sustained without the addition of an electron donor (Dolfing and Tiedje 1986). During the mineralization of 2-CP, benzoate is formed from phenol, the primary dehalogenation product, via sequential carboxylation and dehydroxylation reactions (Becker et al. 1999; Sharak Genthner et al. 1991; Zhang et al. 1990). A second pathway that sometimes occurs during the mineralization of 2-CP and involves benzoate has also been described (Becker et al. 1999). In this alternative pathway, 2-CP undergoes *para*-carboxylation and dehydroxylation to form 3-CB, which is reductively dehalogenated to form benzoate. Benzoate produced during the biodegradation of 2-CP via either pathway is subject to syntrophic conversion to H_2_, acetate, and CO_2_ in the presence of populations that consume the fermentation products (Zhang and Wiegel 1990).

We were interested in tracking the relative activity of populations that mediate syntrophic benzoate fermentation because of the importance of this process in sediment and other anaerobic environments. In particular, we focused on monitoring the activity of *Syntrophus* populations, partly because the DGGE results suggested that the activity of 3CB2-6 decreased with time, whereas 3CB1-4 appeared to increase in abundance (Figure 2).

**Figure 3:**
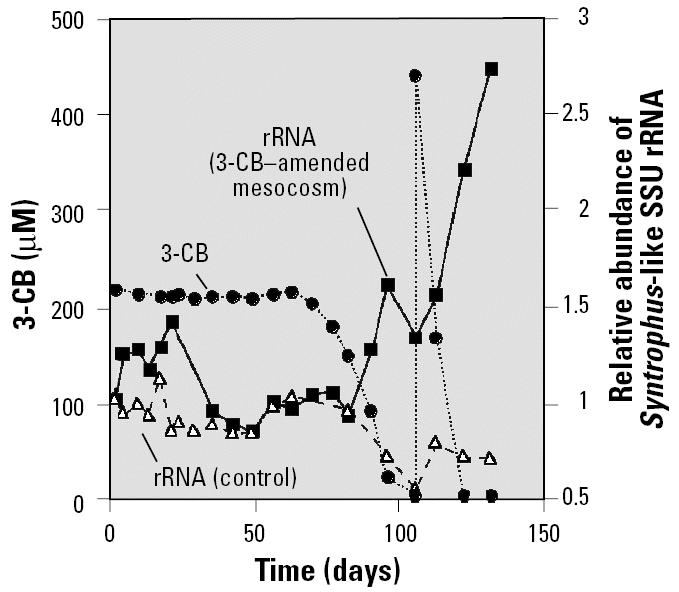
Relative abundance of *Syntrophus*-like SSU rRNA in the 3-CB–amended sediment mesocosm and associated no-substrate control and 3-CB biodegradation in the amended mesocosm. 3-CB concentrations in the sterile control are not shown.

A phylogenetic probe (S-G-Syn-0424-a-A-18), which specifically targets the SSU rRNAs of the three *Syntrophus* species characterized to date and the uncultured population from the sediment community, was designed. Probe S-G-Syn-0424-a-A-18 was used along with an existing probe that targets the SSU rRNA sequences of Archaea to quantify the relative activity of syntrophic benzoate-degrading and methanogenic populations in 2-L mesocosms inoculated with sediment and amended with 3-CB, 2-CP, or no substrate. As shown in Figure 3, transformation of 3-CB in the amended mesocosm followed the pattern observed in the microcosm study and was preceded by an adaptation period of approximately 77 days. During the adaptation period, no 3-CB removal occurred, and the amounts of *Syntrophus*-like rRNA in the 3-CB–amended mesocosm and the no-substrate control fluctuated over time but did not increase significantly overall, compared with the levels initially present at the time of exposure to 3-CB. Once 3-CB biodegradation began, a significant increase in *Syntrophus*-like rRNA levels in the 3-CB–amended mesocosm was observed, and a second increase, which nearly tripled *Syntrophus*-like rRNA levels compared with the initial concentration in this culture, was observed after the addition of a second 3-CB dose. In contrast, during the period of 3-CB biodegradation in the amended mesocosm, *Syntrophus*-like rRNA levels in the no-substrate control decreased. Although benzoate was never detected in the 3-CB–amended mesocosm, it was presumably produced via the reductive dehalogenation of the parent substrate and rapidly consumed by *Syntrophus* species. Thus, the results of the hybridization conducted with mesocosm rRNA extracts support the idea that a *Syntrophus*-like population in the sediment culture was involved in 3-CB biodegradation, as suggested by the DGGE and sequencing analyses performed during the preliminary microcosm experiment. On the basis of previous characterizations of *Syntrophus* species (Jackson et al. 1999; Mountfort et al. 1984; Wallrabenstein et al. 1995), it is likely that this population played a role in fermenting benzoate.

**Figure 4:**
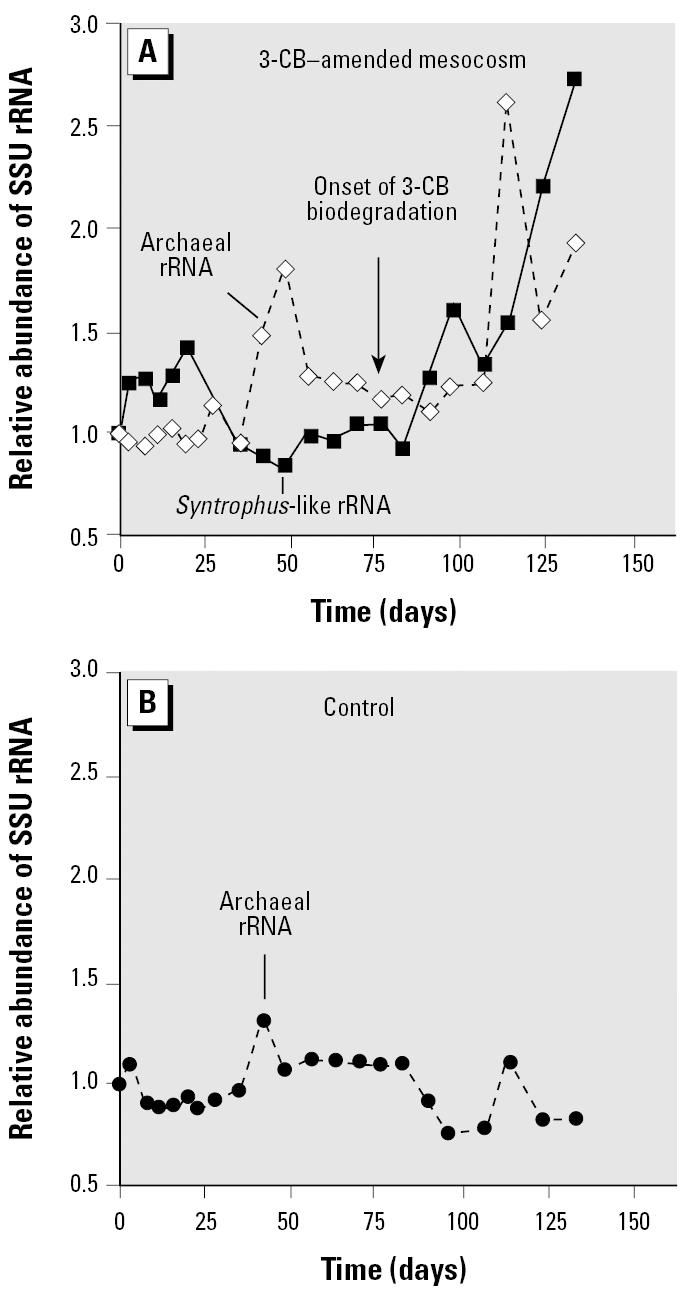
(*A*) Relative abundance of *Syntrophus*-like and archaeal SSU rRNA in the 3-CB–amended sediment mesocosm. (*B*) Relative abundance of archaeal SSU rRNA in the no-substrate sediment control.

Further, the results of the hybridization between rRNA extracted from the 3-CB–amended mesocosm and no-substrate control and the Archaea-specific probe suggested that the activity of the *Syntrophus*-like organisms in the 3-CB–amended mesocosm was sustained by methanogenic populations, which benefited from the production of benzoate-derived H_2_ and/or acetate (Figure 4). In the 3-CB–amended sediment mesocosm, the level of archaeal rRNA, which presumably was derived primarily from methanogens, remained fairly constant during the adaptation period, except for the peak at around day 50, which coincided with the onset of endogenous methane production (Becker 1998). A similar pattern in the level of archaeal rRNA was observed in the no-substrate control during the adaptation period. However, after the onset of 3-CB biodegradation in the amended mesocosm, differences in the activities of methanogenic populations in the two sediment cultures began to emerge. Specifically, in the 3-CB–amended mesocosm, increases in *Syntrophus*-like rRNA were followed by increases in archaeal rRNA. This trend is consistent with the idea that *Syntrophus* and methanogenic populations are members of an syntrophic association in which the *Syntrophus* population, because it increases in abundance and activity, releases greater amounts of electron donors (H_2_ and acetate), which in turn stimulate growth of the methanogens. In contrast, an overall decrease in archaeal rRNA was observed in the no-substrate control after day 77, in the absence of benzoate-derived H_2_ and acetate.

**Figure 5:**
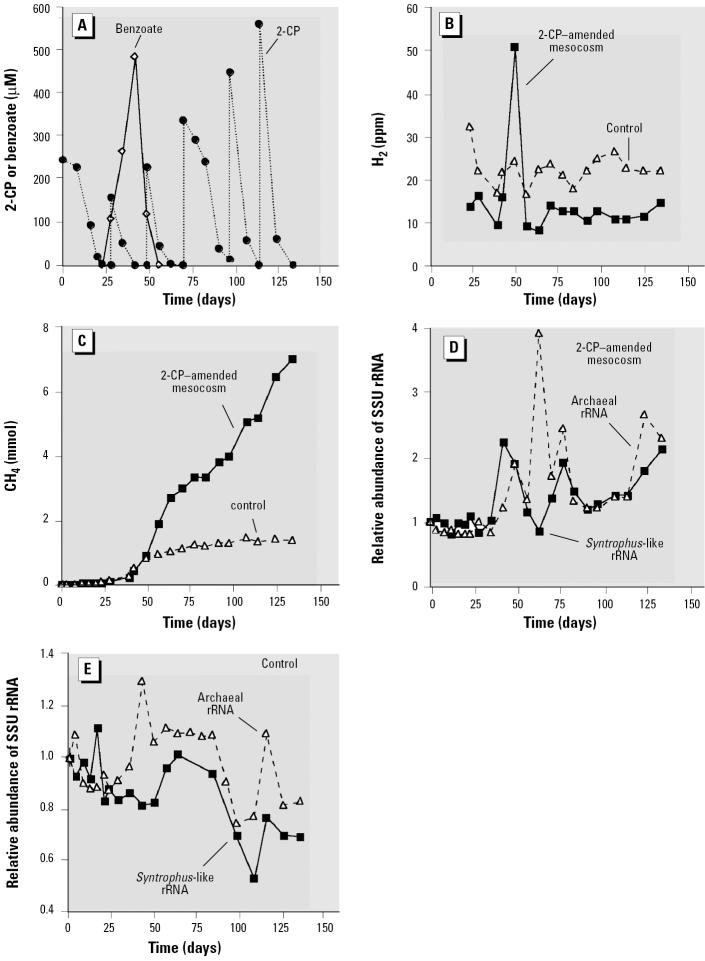
Metabolism of 2-CP and its effects on the activities of syntrophic populations in a 2-CP–amended sediment mesocosm. (*A*) 2-CP transformation and transient accumulation of benzoate in a sediment mesocosm (phenol and 3-CB also accumulated transiently, but are not shown). (*B*) H_2_ levels in the 2-CP–amended mesocosm and associated no-substrate control.(*C*) CH_4_ levels in the 2-CP–amended mesocosm and associated no-substrate control. (*D*) Relative abundance of *Syntrophus*-like and archaeal SSU rRNA in the 2-CP–amended reactor. (*E*) Relative abundance of *Syntrophus*-like and archaeal SSU rRNA in the no-substrate control. 2-CP concentrations in the sterile control are not shown. Adapted from Becker et al. (2001).

Evidence of a syntrophic association between *Syntrophus* and methanogenic populations in the 2-CP–degrading sediment community was also obtained. The mineralization of 2-CP and associated population changes in the sediment community have been reported previously (Becker et al. 2001). Briefly, 2-CP was rapidly removed in the amended mesocosm and was replenished 5 times during the remainder of the approximately 130-day experiment (Figure 5A). In contrast to the studies conducted with 3-CB, benzoate did transiently accumulate in 2-CP–transforming systems. The onset of benzoate fermentation to acetate (Becker et al. 2001) and H_2_ (Figure 5B) occurred by day 49. No hydrogen “burst” was detected in the no-substrate control after the onset of benzoate metabolism in the 2-CP–amended mesocosm. Similarly, after day 49, CH_4_ production increased significantly because of the influx of electron donors in the 2-CP–amended reactor but leveled off in the no-substrate control as endogenous substrates in the sediment were depleted (Figure 5C). The accumulation and depletion of benzoate in the 2-CP–amended mesocosm at around day 49 were paralleled by an increase and decline, respectively, in the level of *Syntrophus*-like rRNA in the amended reactor (Figure 5D). Increases in *Syntrophus*-like rRNA in the amended reactor after day 49 presumably reflect the metabolism of benzoate produced during the biodegradation of 2-CP, although benzoate was not detected in the amended mesocosm after day 53 (Figure 5E). These increases were not observed in the no-substrate control, and overall, the level of *Syntrophus*-like rRNA decreased after day 53 in the control. These data suggest that a *Syntrophus* population also played a key role in the mineralization of 2-CP, that is, fermentation of benzoate. The observed release of H_2_ and increased CH_4_ production in the 2-CP–amended mesocosm, compared with the no-substrate control, are also reflected in the archaeal rRNA levels (Figure 5D). The first archaeal rRNA peak in the 2-CP–degrading mesocosm appeared at approximately the same time that an increase was observed in the 3-CB–amended mesocosm (Figure 4) and the no-substrate control (Figure 5E) and probably reflects, at least partly, methanogenic metabolism of endogenous substrates. However, subsequent archaeal rRNA peaks in the 2-CP–amended reactor either trail or coincide with increases in the level of *Syntrophus*-like rRNA. The coupling of *Syntrophus*-like and archaeal rRNA levels is consistent with the information on the substrates (benzoate and H_2_) and products (CH_4_) and suggests that the metabolisms of the *Syntrophus* and methanogenic population in the 2-CP–degrading sediment community are closely linked.

**Figure 6:**
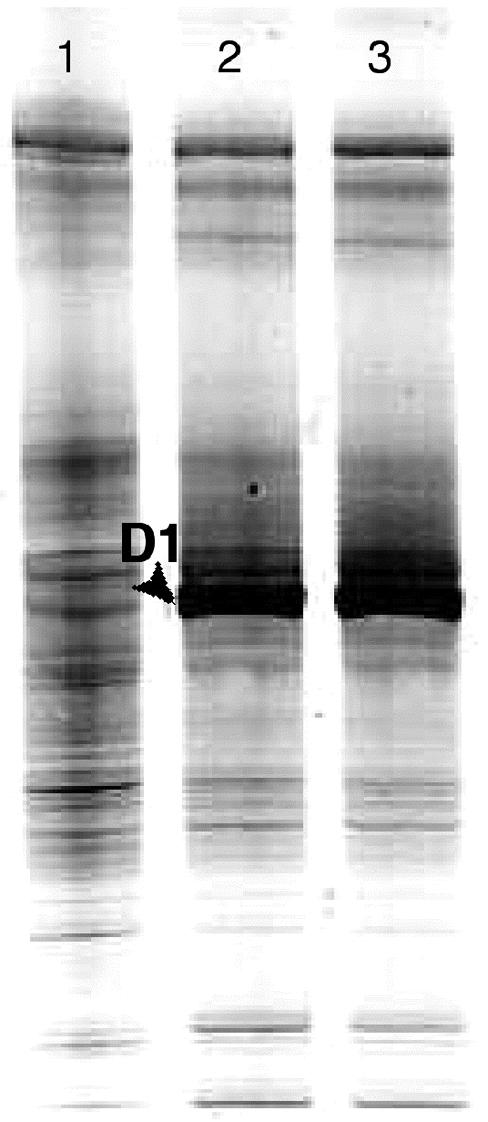
Ethidium bromide–stained DGGE profiles of rDNA fragments of PCR products derived from DNA extracted from mesocosm-scale reactors that were inoculated with digester sludge using a 25–70% denaturant gradient gel. Lane 1 corresponds to community DNA obtained from the no-substrate control after 126 days of incubation in the absence of any added growth substrates. Lanes 2 and 3 correspond to community DNA obtained from duplicate aliquots of the 3-CB–amended digester mesocosm (SBZ1 and SBZ2) that were obtained after ~ 130 days of incubation and 3-CB biodegradation. The aliquots were perturbed with a high dose (~ 14 mM) of benzoate. Benzoate was completely degraded in the aliquots within 40 days and before the DNA was extracted. An arrow indicates the gel portion (SBZ1-D1) that was excised, reamplified, and sequenced. Sequence SBZ1-D1 was submitted to GenBank (http://www.ncbi.nlm.nih.gov/entrez/query.fcgi?db_nucleotide) with the accession number AY646238.

**Figure 7:**
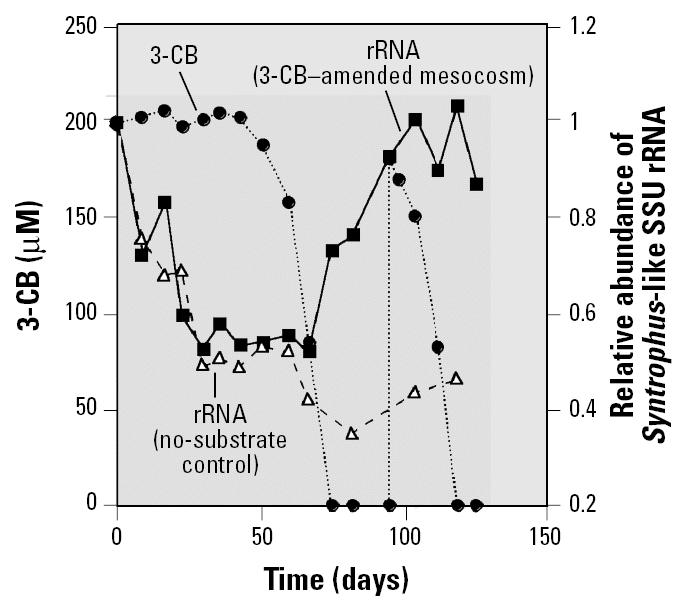
Relative abundance of *Syntrophus*-like SSU rRNA in the 3-CB–amended digester mesocosm and associated no-substrate control and 3-CB biodegradation in the amended mesocosm. 3-CB concentrations in the sterile control are not shown.

Unlike the sediment reactor communities that were studied, the communities in the 3-CB–degrading mesocosm and no-substrate control inoculated with digester sludge remained highly complex over time, as suggested by the DGGE pattern obtained from the no-substrate control at the conclusion of the nearly 130-day experiment (Figure 6). This made it difficult to track and target individual bands in the DGGE patterns obtained from the two digester cultures for further analysis. Consequently, it was also difficult to identify appropriate probe targets for a hybridization involving rRNA extracted from the digester cultures over time. Although DGGE band intensity does not necessarily correspond to population size because of biases caused by extraction efficiencies (Holben 1994) and amplification (von Wintzingerode et al. 1997; Wilson 1997), an attempt was made to facilitate identification of the band(s) associated with benzoate-fermenting population(s) by selectively stimulating their growth. This involved perturbing duplicate aliquots of the 3-CB–amended digester mesocosm contents, which were obtained after two 3-CB additions (200 μM) had been biodegraded in the mesocosm, with a high concentration of benzoate (~ 14 mM). In contrast, reductive dehalogenation of the two 3-CB additions previously exposed the community in the 3-CB–amended digester mesocosm to micro-molar doses of benzoate, which were rapidly consumed. SSU rDNA was extracted and amplified from the digester mesocosm aliquots after the large doses of benzoate were metabolized (within 40 days; data not shown). The DGGE profiles of the benzoate-perturbed digester communities are shown in Figure 6. The intense band that appears in each of these profiles was not detected in DGGE analyses of the 3-CB–degrading digester mesocosm community before perturbation with a high dose of benzoate (not shown) or in the no-substrate control (Figure 6). Sequence SBZ1-D1 was derived from one of these bands (Figure 6) and shares > 99% sequence similarity over 456 bp (excluding three ambiguities) with *Syntrophus aciditrophicus*. On the basis of these results, the *Syntrophus*-specific DNA probe (S-G-Syn-0424-a-A-18) was hybridized with rRNA obtained from the 3-CB–amended digester mesocosm and the associated no-substrate control over time. The results are shown in Figure 7. *Syntrophus*-like rRNA levels in both digester communities decreased significantly during the adaptation period preceding 3-CB biodegradation in the amended reactor. This trend was expected because presumably no benzoate was supplied to either community during this period. *Syntrophus*-like rRNA levels remained low in the no-substrate control until the conclusion of the experiment. In contrast, the onset of 3-CB biodegradation in the amended mesocosm was accompanied by a significant increase in *Syntrophus*-like rRNA levels. The detection of *Syntrophus*-like sequences in the DGGE profiles of the benzoate-perturbed digester community and the results of the hybridization conducted with the *Syntrophus*-specific probe strongly suggest that a *Syntrophus* population played a key role in the biodegradation of chlorinated aromatic compounds in the digester community, as well as in the sediment systems.

The production of CH_4_ in the amended digester mesocosm increased significantly after the onset of 3-CB biodegradation, compared with methanogenesis in the no-substrate control (Becker 1998). The CH_4_ data suggested that methanogens also played an important role in sustaining the biodegradation of the chlorinated substrates by the digester community and benefited from the activity of the *Syntrophus* population. However, the impact of syntrophic benzoate degradation on the activity of methanogenic populations could not be detected by hybridizing the Archaea-specific probe with rRNA extracted from the 3-CB–amended digester mesocosm and no-substrate control over time. Archaeal rRNA levels in the two cultures were of a comparable magnitude and underwent similar temporal changes. Apparently the effect of metabolism of abundant substrates that were endogenous to the digester inocula significantly influenced archaeal SSU rRNA levels and masked the effect of syntrophic benzoate fermentation reflected in the methane levels.

## Conclusions

The results of this study demonstrate that the general approach of using a DNA fingerprinting technique and comparative sequencing to identify potentially important populations in contaminant-degrading communities, followed by SSU rRNA-based hybridizations to quantify changes in the abundance of these populations, can be integrated with analysis of metabolic substrates and products and applied to different complex microbiological systems. Specifically, we demonstrated that it is possible to identify and track populations in syntrophic associations that are based on the fermentation of organic intermediates and are critical for sustaining mineralization of the chlorinated parent compounds in complex anaerobic microbial communities. We anticipate that the analytical approach and methods developed in this study will be useful in the development and monitoring of sustainable engineering strategies for remediation of sites contaminated with chlorinated organic compounds.
